# Secondary Structure of Chloroplast mRNAs In Vivo and In Vitro

**DOI:** 10.3390/plants9030323

**Published:** 2020-03-04

**Authors:** Piotr Gawroński, Aleksandra Pałac, Lars B. Scharff

**Affiliations:** 1 Department of Plant Genetics, Breeding and Biotechnology, Institute of Biology, Warsaw University of Life Sciences, Nowoursynowska 159, 02-776 Warsaw, Poland; 2 Department of Plant and Environmental Sciences, Copenhagen Plant Science Centre, University of Copenhagen, 1871 Frederiksberg C, Denmark

**Keywords:** plastid, Arabidopsis, translation, in vivo RNA secondary structure probing, gene expression

## Abstract

mRNA secondary structure can influence gene expression, e.g., by influencing translation initiation. The probing of in vivo mRNA secondary structures is therefore necessary to understand what determines the efficiency and regulation of gene expression. Here, in vivo mRNA secondary structure was analyzed using dimethyl sulfate (DMS)-MaPseq and compared to in vitro-folded RNA. We used an approach to analyze specific, full-length transcripts. To test this approach, we chose low, medium, and high abundant mRNAs. We included both monocistronic and multicistronic transcripts. Because of the slightly alkaline pH of the chloroplast stroma, we could probe all four nucleotides with DMS. The structural information gained was evaluated using the known structure of the plastid 16S rRNA. This demonstrated that the results obtained for adenosines and cytidines were more reliable than for guanosines and uridines. The majority of mRNAs analyzed were less structured in vivo than in vitro. The in vivo secondary structure of the translation initiation region of most tested genes appears to be optimized for high translation efficiency.

## 1. Introduction

RNA secondary structure in plastids has diverse important functions. It is crucial for ribosome structure and function [1,2,3] as well as for tRNA function [4]. Besides rRNAs and tRNAs, so far, no other functional non-coding RNAs have been described in plastids. Plastid RNase P and signal recognition particle (SRP) lack the RNA component [5,6]. The RNA secondary structure is also important for the function of plastid mRNAs. Start codons can be distinguished from other AUGs by local minima of the mRNA secondary structure [7,8,9]. The mRNA secondary structure of the translation initiation region likely influences the efficiency of translation initiation [8,9], as it is described for *E. coli* [10,11]. In addition, structural changes at the translation initiation region were proposed to be involved in the activation of translation in plastids [12,13,14,15]. This mechanism was also shown to work in synthetic riboswitches in chloroplasts [16]. Furthermore, plastid mRNA secondary structure of coding regions was proposed to influence ribosome pausing [17].

Multiple methods to probe in vivo mRNA secondary structure are available [18,19]. All of them depend on chemical probes that can enter cells and modify nucleotides when they are single-stranded and accessible. One group of probes modifies the bases of nucleotides. Dimethyl sulfate (DMS) methylates N1 of adenosine and N3 of cytidine, and was described to be useful mainly for probing these two nucleotides [20,21]. Glyoxal reacts with single-stranded N1 of adenosine, N3 of cytidine, and N1 of guanosine [22]. 1-ethyl-3-(3-dimethylaminopropyl) carbodiimide (EDC) reacts with single-stranded N1 of guanosine and N3 of uridine [23]. DMS and EDC can be combined to obtain information for all four nucleotides [23]. The same can be achieved using selective 2’-hydroxyl acylation analyzed by primer extension (SHAPE) reagents, e.g., NAI-N_3_. These react with the ribose of nucleotides, more specifically with the 2′-hydroxyl groups in RNA, provided their flexibility is not constrained by base-pairing [24,25]. The classical method to detect the bound probes is to use their ability to terminate reverse transcription and then to determine the termination counts at the different positions [26]. An alternative approach is mutational profiling (MaPseq): An alternative reverse transcriptase (TGIRT) or a specific buffer composition with Mn^2+^ are used, so that reverse transcription does not terminate at the modified nucleotides, but rather incorporates mutations in the cDNA [27,28]. Using reverse transcription termination, only one bound probe per mRNA molecule, the one nearest to the primer, can be detected. In contrast, MaPseq has the advantage that multiple reactions on one mRNA molecule can be detected. MaPseq is used in combination with DMS [28] and SHAPE [27,29]. 

Recently, it was discovered that under alkaline conditions—in contrast to neutral pH—DMS probes all four nucleotides [30]. Mustoe, et al. [30] used an alkaline buffer for DMS probing of *E. coli* and mammalian cells which had a cytosol with neutral pH. However, the pH of the stroma is already slightly alkaline in light, close to pH 8 [31]. Therefore, we expected that in vivo probing of all four nucleotides of chloroplast RNAs should be feasible without the alkaline buffer treatment. Probing *Arabidopsis thaliana* plants, we show that DMS indeed can be used to determine in vivo structural information from all four nucleotides in chloroplasts. However, as evaluated by comparison of the DMS reactivities to a known rRNA structure, the information at adenosines and cytidines is more reliable. Most analyzed mRNAs were less structured in vivo compared to the corresponding in vitro-folded, protein-free RNAs. The secondary structures of most translation initiation regions, especially in vivo, suggest that they are optimized for high translation efficiency.

## 2. Results

Chloroplast in vivo mRNA secondary structure was analyzed in young *Arabidopsis thaliana* plants. As comparison, we analyzed isolated, protein-free RNAs that were allowed to fold in vitro. We probed the mRNA structures with DMS-MaPseq [28]. The plants were incubated with dimethyl sulfate (DMS) which methylates single-stranded and solvent-accessible nucleotides. The methylation and thereby the position of the probe was detected by mutational profiling (MaP), i.e., by comparing the sequence of the cDNA (determined by massively parallel sequencing (seq)) and the known sequence of the transcript. The frequency of mutations provides a quantitative measure of the frequency a specific nucleotide is single-stranded and solvent-accessible in vivo. We chose a selection of plastid transcripts by amplifying their cDNA with specific primers: *clpP* (encoding a proteolytic subunit of the Clp protease), *psaA*/*B*/*rps14* (photosystem I subunits A and B, ribosomal protein uS14c), *psbA* (D1 subunit of photosystem II), *psbD*/*C*/*Z* (photosystem II subunits D2, CP43, and Z), *psbE*/*F*/*L*/*J* (subunits cytochrome *b*_559_ alpha and beta, L, and J of photosystem II), and *rbcL* (large subunit of RuBisCO). We included both high abundant (*psbA*, *rbcL*), medium abundant (*psaA*/*B*/*rps14*, *psbD*/*C*/*Z*, *psbE*/*F*/*L*/*J*), and low abundant (*clpP*) transcripts. In addition, this selection comprises both monocistronic and multicistronic mRNAs. As a control with known structure, we included the plastid 16S rRNA, the RNA component of the 30S subunit of the plastid ribosome [32].

### 2.1. Validation of the Method

**Figure 1:**
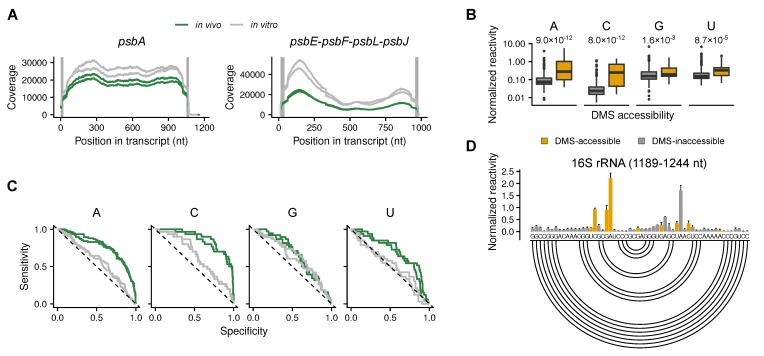
Dimethyl sulfate (DMS) probing of chloroplast RNAs. (**A**) Examples of Illumina reads’ coverage on selected chloroplast transcripts (monocistronic *psbA* and polycistronic *psbE-psbF-psbL-psbJ* are shown) of in vivo samples (green) and in vitro-folded RNA (grey) (the same samples are presented in **C**). Two biological replicates each are shown. Grey, vertical lines depict the regions bound by the primers. These regions are excluded from the further analysis. For the coverage of the other transcripts see Supplemental Figure S1. (**B**) Normalized DMS reactivity at all four nucleotides of the 16S rRNA. The box-plots show that DMS-accessible (i.e., single-stranded and solvent-accessible) nucleotides are more likely to be modified by DMS than DMS-inaccessible (paired or solvent-inaccessible) nucleotides. Statistically significant differences were calculated using the Wilcoxon rank sum test; the resulting p-values are shown. Compare also the detected mutation rate (Supplemental Figure S2B). (**C**) Receiver operating characteristics (ROC) curves for the DMS reactivity profile of chloroplast 16S rRNA for all four nucleotides. (**D**) Normalized DMS reactivity of the in vivo samples of a selected 16S rRNA region (1189–1244 nt). Grey and yellow bars denote DMS-inaccessible and DMS-accessible nucleotides, respectively. Higher normalized DMS reactivity values indicate nucleotides that are more accessible. The secondary structure is presented by an arc-plot (bottom). Base-paired nucleotides are connected by arcs (compare results to [30]).

The coverage for all chosen transcripts was acceptable (Figure 1A, Supplemental Figure S1), and the results are reproducible (Supplemental Figure S2A). As expected, adenosines and cytidines can be probed with DMS (Supplemental Figure S2B). Accessible (single-stranded and solvent-accessible) and inaccessible nucleotides of the 16S rRNA, as determined by the known structure [32], can be clearly distinguished by DMS-MaPseq (Figure 1B). Compared to the known structure of the 16S rRNA, the probing of adenosines and cytidines revealed a clear structure signal, which in vivo was clearly different from the in vitro-folded, protein-free rRNA (Figure 1C). Mustoe et al. [30] report that in slightly alkaline conditions DMS can also be used to probe guanosines and uridines. As the chloroplast stroma is slightly alkaline in vivo [31], we additionally compared the DMS reactivity of these nucleotides with the known structure of the 16S rRNA [32]. The structure signals for guanosines and uridines were weaker than the ones for adenosines and cytidines (Figure 1B,C) but still informative. As an example of the DMS reactivities, a region of the 16S rRNA is shown, including information about which nucleotides are accessible to DMS (Figure 1D).

### 2.2. Analysis of the Secondary Structure of Start Codons

**Figure 2:**
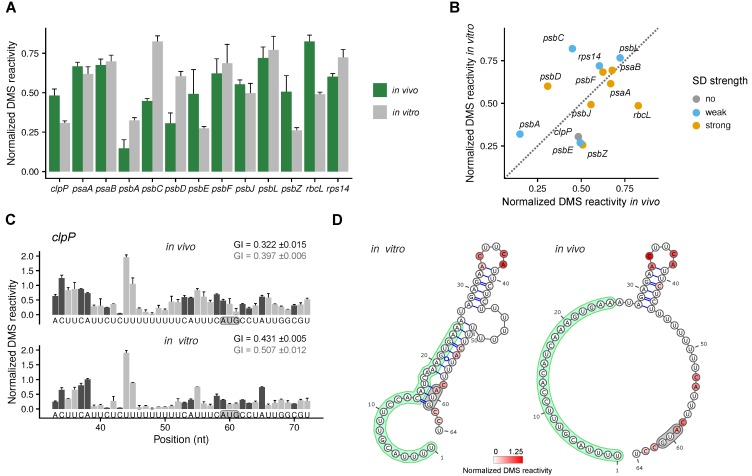
Analysis of mRNA secondary structure of start codons. (**A**) Average normalized DMS reactivities at the start codon in in vivo samples (green) and in vitro-folded RNA (grey) (only adenosines were analyzed). Higher normalized DMS reactivity values indicate start codons that are more accessible. (**B**) Comparison of the average normalized DMS reactivity at the start codon between in vitro-folded RNA and in vivo. The color code marks genes with strong Shine–Dalgarno (SD) sequences (hybridization to the anti-SD of the 16S rRNA < −6 kcal mol^−1^, green), weak SD (>−6–< 0 kcal mol^−1^, blue), and no SD (>0 kcal mol^−1^, grey). (**C**) Normalized DMS reactivities at the translation initiation region of *clpP*. The dark grey bars represent the more reliable probing of adenosines and cytidines (compare to Figure 1B,C), light grey is the less reliable probing of guanosines and uridines. The start codon is marked with a grey background. The sequence covered by the primer used to amplify the cDNA (1–32 of the *clpP* mRNA) does not contain any information about the mRNA secondary structure and is therefore not shown. As measure of the secondary structure of the whole translation initiation region, the Gini index (GI) is given for adenosines/cytidines (black) and all four nucleotides (grey). A value close to 0 indicates a low amount of structure, a value close to 1 a high amount of structure. (**D**) Models of the secondary structure of the translation initiation region of *clpP* in vivo and in vitro (see Supplemental Figure S3 for a prediction of the structure of the *clpP* transcript). The color code indicates the DMS reactivities at adenosines and cytidines. The green part is the footprint of the putative RNA binding protein [33]. The start codon is marked with a grey background.

About 30% of Arabidopsis plastid genes do not have a Shine–Dalgarno (SD) sequence to determine their start codon (Supplemental Table S1). For these genes, local minima of secondary structure were proposed to determine their start codon and thereby their translation efficiency [7]. *clpP* is an example of a gene without SD [8,9]. The DMS reactivity at the start codon was higher in vivo compared to the in vitro-folded, protein-free RNA (Figure 2A–C). This strongly indicates that the start codon is more accessible to ribosomes in vivo, presumably as the structure is modified by an RNA binding protein. Ruwe et al. [33] detected a footprint of a RNA binding protein in the *clpP* 5′ UTR, whose binding would indeed prevent the formation of a stem loop and thereby increase the accessibility of the start codon (Figure 2D). In our gene set, we found three other genes whose start codons had a higher accessibility in vivo than in vitro: *psbE* (with a weak SD), *psbZ* and *rbcL* (both with strong SDs) (Figure 2A,B). Six genes had highly accessible start codons both in vivo and in vitro. Among them were two genes, *psbL* and *rps14*, with weak SDs. The remaining four genes possess strong SDs: *psaA* and *psaB* as well as *psbF* and *psbJ*. There were three cases in which the start codon accessibility was higher in vitro than in vivo: *psbA*, *psbC*, and *psbD* (Figure 2A,B). *psbD* has a strong SD, and translation of its mRNA is unlikely to depend on an accessible start codon. In contrast, *psbA* has only a weak SD and would be more dependent on an accessible start codon. In the low light conditions analyzed (see Section 4.1.), *psbA* translation efficiency is expected to be low [34]. According to the annotation, *psbC* possesses a start codon with a very weak SD, and translation would be expected to depend on mRNA secondary structure. However, in *Nicotiana tabacum*, *psbC* has a GUG as start codon. This GUG is conserved in Arabidopsis and would be determined by a strong SD (Supplemental Table S1). It is not known, if the annotated AUG or the alternative GUG is used in vivo. However, the report of a footprint of a RNA binding protein that covers the annotated AUG start codon [33] suggests that the downstream GUG could be used in vivo in Arabidopsis. Most differences of the secondary structure of the start codon between in vivo and in vitro were detected for monocistronic genes or the first gene in an operon. This could indicate that it is more likely that RNA binding proteins are found at or near the 5′ ends of transcripts. Exceptions were *psbC* (which also exists as monocistronic mRNA [35,36]) and *psbZ*. Both are located downstream of the first gene of the operon, but their start codon accessibility also differs in vivo and in vitro. In most cases, the start codon is either already accessible in in vitro-folded RNA or at least accessible in vivo. This is in agreement with the assumption that most start codons are part of mRNA secondary structures optimized for high translation efficiency.

### 2.3. Analysis of the Secondary Structures of Shine-Dalgarno Sequences and Translation Initiation Regions

**Figure 3:**
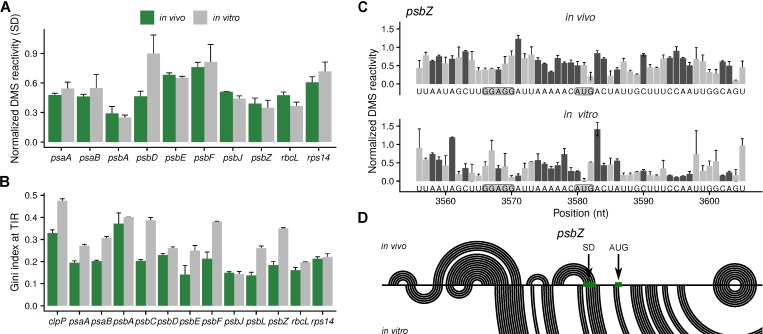
Analysis of mRNA secondary structure of Shine-–Dalgarno sequences (SD) and translation initiation regions (TIR). (**A**) Average normalized DMS reactivities at the Shine–Dalgarno sequences in in vivo samples (green) and in vitro-folded RNA (grey) (adenosines and cytidines were analyzed). Only genes with SD are included (Supplemental Table S1). Higher normalized DMS reactivity values indicate nucleotides that are more accessible. (**B**) Gini index of the translation initiation regions (−25 to +5 relative to the first nucleotide of the start codon). A value close to 0 indicates a low amount of structure, a value close to 1 a high amount of structure. (**C**) The normalized DMS reactivities at the translation initiation region of *psbZ*. The dark grey bars represent the more reliable probing of adenosines and cytidines (compare to Figure 1B,C), light grey is the less reliable probing of guanosines and uridines. The Shine–Dalgarno sequence (GGAGG) and the start codon (AUG) are marked with a grey background. (**D**) Predicted mRNA secondary structures of the *psbZ* translation initiation region in vivo and in vitro presented as arc-plots using DMS reactivities (at adenosines and cytidines) as constrains. The positions of the Shine–Dalgarno sequence (SD) and the start codon (AUG) are marked (compare also Figure 2A for the DMS reactivity at the start codon).

The majority of genes in the Arabidopsis plastome have Shine–Dalgarno sequences (SDs) that can interact with the anti-Shine–Dalgarno sequence at the 3′ end of the 16S rRNA, and thus determine their start codons (Supplemental Table S1). For most analyzed genes, the in vivo secondary structure of SDs was unaltered compared to in vitro-folded RNA (Figure 3A). An exception was *psbD*, whose SD was more accessible in vitro. Furthermore, we analyzed the structure changes in the translation initiation region (defined as nucleotides −25 to +5 relative to the start codon). Using the Gini index as a measure of mRNA secondary structure, for most genes, we found less structured translation initiation regions in vivo compared to in vitro-folded, protein-less mRNA (Figure 3B). An example is the translation initiation region of *psbZ* (Figure 3C,D). This indicates that the translation initiation regions have more favorable secondary structures in vivo, i.e., structures that facilitate the access of the ribosome.

### 2.4. Analysis of the Secondary Structures of Coding Regions

**Figure 4:**
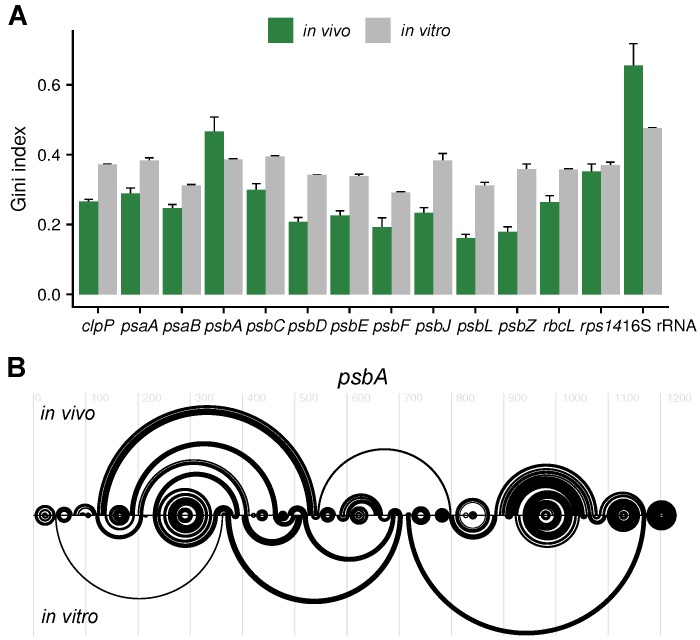
Analysis of mRNA secondary structure of coding regions. (**A**) Gini index of the coding regions for in vivo samples (green) and in vitro-folded RNA (grey). A value close to 0 indicates a low amount of structure, a value close to 1 a high amount of structure. Included is the 16S rRNA that was used as control with known structure (see Figure 1B–D). See also the normalized DMS reactivities of the complete RNAs (Supplemental Figure S4). (**B**) Predicted mRNA secondary structure of *psbA* in vivo and in vitro presented as arc-plots using DMS reactivities (at adenosines and cytidines) as constrains. The full-length transcript was used for the predictions.

The secondary structure of the coding region is expected to differ between in vivo conditions, i.e., with bound ribosomes and RNA binding proteins, and in vitro conditions without any proteins. Most analyzed coding regions were less structured in vivo, as measured by Gini index (Figure 4A), probably due to partial unfolding by elongating ribosomes. In contrast, the Gini index for the 16S rRNA (*rrn16*) indicates that it is more structured in vivo. Likely, bound ribosomal proteins and the compact rRNA structure reduce the solvent accessibility, i.e., the accessibility for DMS, and thereby lower the DMS reactivity. Deviating from the pattern of the other protein-encoding genes (Figure 4A), the coding region of *psbA* is more structured in vivo (as measured by the Gini index) (Figure 4A). Furthermore, it is more structured in vivo than all other analyzed coding regions. *psbA* translation efficiency is expected to be low in low light [34]. Either the *psbA* coding region is more structured in low light or more RNA binding proteins are bound to it. The structure predictions based on the DMS probing of the *psbA* mRNA differ strikingly in vivo and in vitro (Figure 4B).

### 2.5. The Secondary Structure of An Antisense tRNA Sequence in the psbD/C/Z Operon

**Figure 5:**
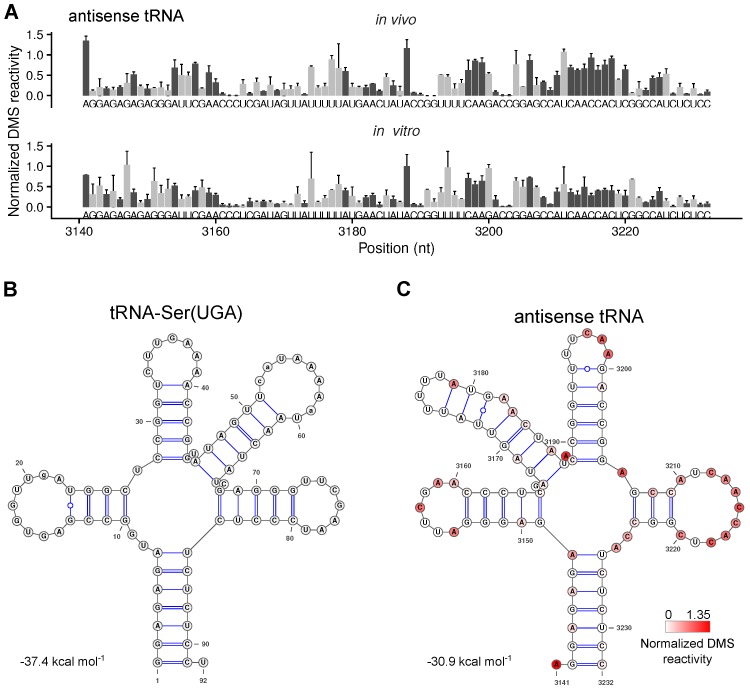
Analysis of RNA secondary structure of an antisense tRNA between *psbC* and *psbZ*. The *trnS-UGA* gene encoding for the tRNA-Ser(UGA) is localized on the opposite strand of the *psbD*/*C*/*Z* operon, so that an antisense copy of the tRNA is included in the operon between *psbC* and *psbZ*. (**A)** Normalized DMS reactivities at the antisense tRNA sequence of in vivo-and in vitro-folded samples. A value close to 0 indicates a low amount of structure, a value close to 1 a high amount of structure. The dark grey bars represent the more reliable probing of adenosines and cytidines (compared to Figure 1B,C), light grey is the less reliable probing of guanosines and uridines. (**B**) The secondary structure of tRNA-Ser(UGA) is predicted by tRNAscan-SE v. 2.0 [39,40]. (**C**) Predicted mRNA secondary structure of the antisense tRNA sequence using DMS reactivities of the in vivo samples as constrains. The color code indicates the normalized DMS reactivities at adenosines and cytidines.

The *psbD*/*C*/*Z* operon contains an oddity: tRNA-Ser(UGA) is encoded on the opposite strand between *psbC* and *psbZ*. Most tRNA genes in plastomes are located between transcription units; often they are separating them from each other. The *trnS-UGA* gene is a notable exception. The processing of the *psbD*/*C*/*Z* operon is very complex and includes transcripts spanning the location of *trnS-UGA*, which is therefore transcribed as reverse complement. The tricistronic and dicistronic mRNA species including *psbZ* downstream of the antisense tRNA accumulate to lower levels than the upstream dicistronic *psbD*/*C* and monocistronic *psbC* species [35,36,37,38] (see also Supplemental Figure S1). We wondered if the antisense tRNA sequence folds into a tRNA-like structure, which could trigger processing of the antisense tRNA sequence by the RNases responsible for tRNA processing. The in vivo secondary structure of the antisense sequence is indeed tRNA-like (Figure 5A–C) and could thus be responsible for processing between *psbC* and *psbZ*. There is a footprint of a RNA binding protein downstream of *psbC* [33] that could stabilize the 3′ end of the dicistronic *psbD*/*C* and the monocistronic *psbC*. However, no protein or its footprint have been described that could stabilize the 5′ end of a monocistronic *psbZ*, which could explain why this monocistronic species was not reported [35,36,37,38].

## 3. Discussion

We determined the in vivo and the in vitro, protein-free mRNA secondary structure of a selection of chloroplast mRNAs using DMS-MaPseq in combination with specific primers. To validate the approach, the DMS reactivities at the 16S rRNA were compared to its known structure (Figure 1). As expected, the structure signals at adenosines and cytidines were good (Figure 1C). Because of the slightly alkaline pH in the chloroplast stroma [31], information about the secondary structure could also be detected for guanosines and uridines, but, compared to a known structure, the reliability is lower than for adenosines and cytidines (Figure 1C). In contrast to probing of organisms with neutral pH in the cytosol [30], the leaf chloroplast RNA could be directly probed in vivo without incubating the plants in alkaline buffer. It is possible that a treatment with an alkaline bicine buffer would improve the structural signal for guanosines and uridines. Using specific primers for the amplification of cDNAs before sequencing, we gained sufficient coverage to probe high abundant as well as low abundant mRNAs (Figure 2, Figure 3 and Figure 4, Supplemental Figure S1). The drawback of this approach is that there is no structural information for the extreme 5′ and 3′ end of the mRNAs, as these are masked by the used primers. Furthermore, the read length of the chosen sequencing method requires fragmentation of the amplified cDNAs. Therefore, it was not possible to assign DMS reactivities to a specific subspecies of mRNA. This would be interesting for mRNAs transcribed from different promoters or with different 5′ end processing as well as for genes that are present on monocistronic and multicistronic mRNAs [37,41,42]. Different 5′ ends of a given mRNA species could influence the secondary structure, especially the structure of the translation initiation regions.

DMS probes single-stranded and accessible nucleotides [20]. It is optimal for analyzing if specific cis-elements, e.g., Shine–Dalgarno sequences and start codons, are accessible. High DMS reactivity at a nucleotide indicates that it is single-stranded in the majority or all analyzed mRNA molecules. However, it is difficult to distinguish if low DMS reactivity is caused by double-stranded nucleotides, by bound proteins or low accessibility for DMS in a compact secondary structure [43,44].

DMS methylates nucleotides, and the modified nucleotides are detected by their ability to terminate reverse transcription or cause mutations in the cDNA [28,43]. RNA is also methylated in vivo. Chloroplast mRNAs are methylated at N6 of adenosines (m^6^A) [45], and plastid rRNAs and tRNAs at C5 of cytidines (m^5^C) [46], but both RNA modifications do not influence reverse transcription. N1 methylation of adenosines (m^1^A) that was detected in very low abundance in human mRNAs [47] would be identical to methylation of adenosines by DMS. However, this modification is not known to exist in plastids. Pseudouridines (Ψ) were detected in chloroplasts mRNAs [48], but also do not influence reverse transcription. RNA editing, and conversion of cytidines into uridines [49], cannot be distinguished from DMS-caused mutations in reverse transcription. Therefore, we excluded known editing sites in chloroplast mRNAs and known modified nucleotides of the 16S rRNA from our analysis (see Section 4.5.).

For most genes, the in vivo and in vitro RNA secondary structure differed in both the translation initiation region and the coding region (Figure 2, Figure 3 and Figure 4). There are multiple possible causes for such differences. The folding of freshly transcribed mRNA in vivo is likely polar, i.e., it starts at the 5′ end, whereas already transcribed RNAs that are allowed to refold in vitro are not expected to have such a bias. *In vivo*, multiple RNA binding proteins influence the mRNA secondary structure. This includes proteins protecting the 5′ and 3′ ends, proteins regulating translation, RNA chaperones, proteins protecting untranslated mRNAs, and—likely only transiently—proteins responsible for splicing and RNA editing [49,50,51,52,53,54]. However, RNA secondary structure, on the other hand, influences also the binding of RNA binding proteins [55]. Translating ribosomes unfold mRNAs, mainly in the coding regions, as described for bacteria [11,56]. Furthermore, different ion compositions of the stroma, e.g., rising magnesium concentrations [57] during development, could influence mRNA folding.

## 4. Materials and Methods

### 4.1. Plant Material

*Arabidopsis thaliana* wild-type (ecotype Col-0) plants were grown in Jiffy pots (Jiffy Products) for 17–18 days at 22 °C and 150 µE m^−2^ s^−1^ in long-day conditions (16 h day/8 h night). For the in vivo dimethyl sulfate (DMS) modification, the plants were transferred to a fume hood where they were kept for 1 h in dim light (~10 µE m^−2^ s^−1^) and then treated with DMS (see Section 4.2.). For in vitro DMS modification, after 1 h in dim light, plants were frozen in liquid nitrogen and kept in −80 °C. For in vivo and in vitro samples, two biological samples were used, each was a pool of at least three rosettes to obtain the required amount of material for RNA isolation.

### 4.2. RNA Structure Probing with DMS

For in vivo probing, decapitated rosettes were collected into 10 mL of DMS reaction buffer (100 mM KCl, 40 mM HEPES pH 7.5, 0.5 mM MgCl_2_) in 50 mL tubes. DMS (Sigma-Aldrich) was added to a concentration of 5% (w/v), and the reaction was performed at 24–25 °C for 6 min. The reaction was stopped by adding 20 mL of ice-cold 30% β-mercaptoethanol (Sigma–Aldrich) and incubating for 1 min on ice. Afterwards the liquid was removed, the plants were washed twice with distilled water, frozen in liquid nitrogen, and stored in −80 °C. Since prolonged incubation or high DMS concentration may cause RNA degradation, especially in young plants [58], we experimentally determined the used DMS concentration and incubation time in order to obtain high quality, non-degraded RNA.

RNA was extracted from in vivo DMS treated plants and from untreated plants for in vitro DMS probing using the Spectrum Plant Total RNA Kit (Sigma–Aldrich). DNA was removed using the Turbo DNA-free kit (Thermo Fisher Scientific).

For in vitro DMS probing, 5 µg (20 µL) of RNA in water was heat-denatured for 2 min at 95 °C and quickly transferred to ice. 80 µL of DMS reaction buffer (100 mM KCl, 40 mM HEPES pH 7.5, 0.5 mM MgCl_2_) and 100 U of Murine Rnase Inhibitor (NEB) were added, followed by incubation with mixing at 25 °C for 5 min. Next, DMS was added to the final concentration of 5%, and samples were incubated for 6 min at 25 °C with gentle mixing. The reaction was terminated by adding 200 µL of ice-cold 30% β-mercaptoethanol and incubating for 1 min on ice. RNA was recovered by ethanol precipitation.

### 4.3. cDNA Synthesis

Prior to cDNA synthesis, RT primers (Table S1) were mixed equimolarly, and the concentration of each primer in the mix was 2 µM. 1 µL of RT primer mix was annealed to 2 µg of RNA in 5.9 µL water at 65 °C and immediately placed on ice. Next, 100 units of TGIRT-III (InGex) reverse transcriptase in TGIRT buffer (50 mM Tris-HCl pH 8.3, 75 mM KCl, 3 mM MgCl_2_), 0.5 µL of 100 mM freshly prepared dithiothreitol, and 4 units of Murine RNase Inhibitor (NEB) were added to primer annealed RNA, and pre-incubation was conducted at 25 °C for 30 min. Reverse transcription was initiated by addition of 1 µL of 10 mM dNTPs and performed for 2 h at 57 °C. The final volume of the reaction was 10 µL. RNA was removed by adding 5 units of RNase H (NEB) and incubating for 20 min at 37 °C. RNase H was inactivated by 20min incubation at 65 °C. cDNA was purified using 2.5X strength Ampure XP beads (Beckman Coulter) and resuspended in 100 µL of RNase-free water (EURx).

### 4.4. PCR Amplification

The aim of this experiment was to analyze structures of whole chloroplast transcripts that are of different lengths and expression levels. As we aimed to obtain equally good sequencing coverage for both highly and lowly expressed genes, we decided to use an approach where full transcripts are amplified with single or multiple pairs of gene specific primers (Supplemental Table S2). PCRs used 1 µL of cDNA, 0.4 U of Q5^®^ Hot Start High-Fidelity DNA Polymerase (NEB), 1 × Q5 Reaction Buffer, 200 µmol/L dNTPs and 0.5 µmol/L of each primer in a final volume of 20 µL. PCRs were performed as follows: 98 °C for 30 s, 23–31 cycles (depending on the gene, see Supplemental Table S3) of [98 °C for 15 s, 65 °C for 5 min]. PCR products were clean-up using 1.8X strength Ampure XP beads (Beckman Coulter). Amplification specificity was evaluated using agarose gel electrophoresis. DNA concentration and purity were evaluated using NanoDrop™ 2000c (ThermoFisher). PCR products from the same samples were mixed equimolarly and were subsequently used for sequencing library preparation using NEBNext^®^ Ultra™ II FS DNA Library Prep Kit for Illumina (NEB) according to the protocol that included a step to fragment the cDNA to 150–250 bp. Libraries were sequenced with an Illumina MiSeq instrument using 2 × 300 paired-end sequencing (Oligo.pl).

### 4.5. Data Analysis

Sequencing reads were trimmed using Trim Galore! (version 0.6.5; http://www.bioinformatics.babraham.ac.uk/projects/trim_galore/) using following settings: --paired -j 8 --three_prime_clip_R1 5 --three_prime_clip_R2 50 --fastqc --quality 35. Next, trimmed reads were mapped to chloroplast transcripts using bowtie2 (version 2.3.4.1) [59] with settings: -local–very-sensitive-local. Mutation frequencies for analyzed transcripts were calculated using the pileup function from the Rsamtools package [60]⁠. For further analysis, substitutions and deletions of nucleotides with coverage of at least 2500 mapped reads and not covered by PCR primers were used. In addition, all RNA editing sites and modified nucleotides in the 16S rRNA were removed from analysis. Raw DMS reactivities (mutation rates, see Figure S2B) were normalized as described by Mustoe et al. [30]⁠. Briefly, A/C and G/U reactivities were normalized separately because DMS reactivity at G/U is significantly lower than at A/C (Figure S2B). Normalization was done by dividing the reactivities (mutation rates) of each transcript by the average reactivity of the 90th–99th percentile of the most highly reactive nucleotides of the same transcript. Next, extremely high values were removed by transcript-wise 99% winsorization. The obtained values were named “normalized DMS reactivity” and have been presented in plots as mean ±SEM. To assess the quality of the obtained data, we performed a receiver operating characteristic (ROC) analysis for individual nucleotides of the 16S rRNA using the pROC package [61]⁠. True positive nucleotides were defined as nucleotides that were both unpaired and solvent-accessible in the crystal structure model [32]⁠. Gini indexes were calculated using normalized DMS reactivities at As and Cs (except where indicated otherwise) using the gini.index function from the lawstat package. Gini indexes are presented in the plots as mean ±SEM.

### 4.6. 70S Structure Analysis

The crystal structure of the chloroplast 70S ribosome [32]⁠ was downloaded from PDB (https://www.rcsb.org/, entry 5X8P). Surface residues (i.e., solvent accessible) were calculated in PyMOL using the FindSurfaceResidues module. Residues with an area >2.5 Å^2^ were considered as solvent accessible.

### 4.7. RNA Structure Prediction

RNAstructure (version 6.2) [62]⁠ was used for structure modeling of full length transcripts. Only reactivities of As and Cs were used as soft constrains in the Fold program, and a single minimum free energy structure is reported. Fold program parameters were as follows: -md 500 -t 298.15. Predicted structures were visualized using R4RNA [63]⁠ and VARNA [64]⁠.

### 4.8. Prediction of Shine-Dalgarno Sequences

The strength of the interaction of the Shine–Dalgarno sequence and the anti-SD CCUCCU of the 16S rRNA was determined by in silico hybridizing the anti-SD to nucleotides −22 to −2 of each 5′ UTR at 20 °C using Free2bind [65].

## Supplementary Materials

The following are available online at https://www.mdpi.com/2223-7747/9/3/323/s1, Supplemental Figure S1 Illumina reads’ coverage of chloroplast transcripts. Supplemental Figure S2 Reproducibility of DMS reactivities. Supplemental Figure S3 Predicted mRNA secondary structures of *clpP* in vivo and in vitro. Supplemental Figure S4 Comparison of normalized DMS reactivities (of As and Cs) at all analyzed RNAs in vivo and in vitro. Supplemental Table S1 Strength of the interaction of the Shine-Dalgarno sequence and the anti-SD of the 16S rRNA. Supplemental Table S2. List of primers used in this study. Supplemental Table S3. Table showing number of additional PCR cycles relative to *psbA*.

## References

[1] Ehrenberg M. (2009). Scientific background on the Nobel Prize in Chemistry 2009 structure and function of the ribosome. R. Swedish Acad. Sci..

[2] Bieri P., Leibundgut M., Saurer M., Boehringer D., Ban N. (2017). The complete structure of the chloroplast 70S ribosome in complex with translation factor pY. EMBO J..

[3] Perez Boerema A., Aibara S., Paul B., Tobiasson V., Kimanius D., Forsberg B.O., Wallden K., Lindahl E., Amunts A. (2018). Structure of the chloroplast ribosome with chl-RRF and hibernation-promoting factor. Nat. Plants.

[4] Giegé R., Jühling F., Pütz J., Stadler P., Sauter C., Florentz C. (2012). Structure of transfer RNAs: Similarity and variability. Wiley Interdiscip. Rev. RNA.

[5] Pinker F., Bonnard G., Gobert A., Gutmann B., Hammani K., Sauter C., Gegenheimer P.A., Giegé P. (2013). PPR proteins shed a new light on RNase P biology. RNA Biol..

[6] Ziehe D., Dünschede B., Schünemann D. (2017). From bacteria to chloroplasts: Evolution of the chloroplast SRP system. Biol. Chem..

[7] Scharff L.B., Childs L., Walther D., Bock R. (2011). Local absence of secondary structure permits translation of mRNAs that lack ribosome-binding sites. PLoS Genet..

[8] Zhang J., Ruf S., Hasse C., Childs L., Scharff L.B., Bock R. (2012). Identification of cis-elements conferring high levels of gene expression in non-green plastids. Plant J..

[9] Scharff L.B., Ehrnthaler M., Janowski M., Childs L.H., Hasse C., Gremmels J., Ruf S., Zoschke R., Bock R. (2017). Shine-dalgarno sequences play an essential role in the translation of plastid mRNAs in tobacco. Plant Cell.

[10] Kudla G., Murray A.W., Tollervey D., Plotkin J.B. (2009). Coding-sequence determinants of gene expression in *Escherichia coli*. Science.

[11] Mustoe A.M., Busan S., Rice G.M., Hajdin C.E., Peterson B.K., Ruda V.M., Kubica N., Nutiu R., Baryza J.L., Weeks K.M. (2018). Pervasive regulatory functions of mrna structure revealed by high-resolution SHAPE probing. Cell.

[12] Stampacchia O., Girard-Bascou J., Zanasco J.L., Zerges W., Bennoun P., Rochaix J.-D. (1997). A nuclear-encoded function essential for translation of the chloroplast psaB mRNA in chlamydomonas. Plant Cell.

[13] Klinkert B., Elles I., Nickelsen J. (2006). Translation of chloroplast psbD mRNA in Chlamydomonas is controlled by a secondary RNA structure blocking the AUG start codon. Nucleic Acids Res..

[14] Prikryl J., Rojas M., Schuster G., Barkan A. (2011). Mechanism of RNA stabilization and translational activation by a pentatricopeptide repeat protein. Proc. Natl. Acad. Sci. USA.

[15] Hammani K., Cook W.B., Barkan A. (2012). RNA binding and RNA remodeling activities of the half-a-tetratricopeptide (HAT) protein HCF107 underlie its effects on gene expression. Proc. Natl. Acad. Sci. USA.

[16] Verhounig A., Karcher D., Bock R. (2010). Inducible gene expression from the plastid genome by a synthetic riboswitch. Proc. Natl. Acad. Sci. USA.

[17] Gawroński P., Jensen P.E., Karpiński S., Leister D., Scharff L.B. (2018). Pausing of chloroplast ribosomes is induced by multiple features and is linked to the assembly of photosynthetic complexes. Plant Physiol..

[18] Strobel E.J., Yu A.M., Lucks J.B. (2018). High-throughput determination of RNA structures. Nat. Rev. Genet..

[19] Mitchell D., Assmann S.M., Bevilacqua P.C. (2019). Probing RNA structure in vivo. Curr. Opin. Struct. Biol..

[20] Wells S.E., Hughes J.M., Igel A.H., Ares M. (2000). Use of dimethyl sulfate to probe RNA structure in vivo. Methods Enzymol..

[21] Ding Y., Tang Y., Kwok C.K., Zhang Y., Bevilacqua P.C., Assmann S.M. (2014). In vivo genome-wide profiling of RNA secondary structure reveals novel regulatory features. Nature.

[22] Mitchell D., Ritchey L.E., Park H., Babitzke P., Assmann S.M., Bevilacqua P.C. (2018). Glyoxals as in vivo RNA structural probes of guanine base-pairing. RNA.

[23] Wang P.Y., Sexton A.N., Culligan W.J., Simon M.D. (2019). Carbodiimide reagents for the chemical probing of RNA structure in cells. RNA.

[24] Merino E.J., Wilkinson K.A., Coughlan J.L., Weeks K.M. (2005). RNA structure analysis at single nucleotide resolution by Selective 2′-Hydroxyl Acylation and Primer Extension (SHAPE). J. Am. Chem. Soc..

[25] McGinnis J.L., Dunkle J.A., Cate J.H.D., Weeks K.M. (2012). The mechanisms of RNA SHAPE chemistry. J. Am. Chem. Soc..

[26] Spitale R.C., Crisalli P., Flynn R.A., Torre E.A., Kool E.T., Chang H.Y. (2013). RNA SHAPE analysis in living cells. Nat. Chem. Biol..

[27] Siegfried N.A., Busan S., Rice G.M., Nelson J.A.E., Weeks K.M. (2014). RNA motif discovery by SHAPE and mutational profiling (SHAPE-MaP). Nat. Methods.

[28] Zubradt M., Gupta P., Persad S., Lambowitz A.M., Weissman J.S., Rouskin S. (2017). DMS-MaPseq for genome-wide or targeted RNA structure probing in vivo. Nat. Methods.

[29] Smola M.J., Weeks K.M. (2018). In-cell RNA structure probing with SHAPE-MaP. Nat. Protoc..

[30] Mustoe A.M., Lama N.N., Irving P.S., Olson S.W., Weeks K.M. (2019). RNA base-pairing complexity in living cells visualized by correlated chemical probing. Proc. Natl. Acad. Sci. USA.

[31] Su P.-H., Lai Y.-H. (2017). A reliable and non-destructive method for monitoring the stromal pH in isolated chloroplasts using a fluorescent pH probe. Front. Plant Sci..

[32] Ahmed T., Shi J., Bhushan S. (2017). Unique localization of the plastid-specific ribosomal proteins in the chloroplast ribosome small subunit provides mechanistic insights into the chloroplastic translation. Nucleic Acids Res..

[33] Ruwe H., Wang G., Gusewski S., Schmitz-Linneweber C. (2016). Systematic analysis of plant mitochondrial and chloroplast small RNAs suggests organelle-specific mRNA stabilization mechanisms. Nucleic Acids Res..

[34] Schuster M., Gao Y., Schöttler M.A., Bock R., Zoschke R. (2020). Limited responsiveness of chloroplast gene expression during acclimation to high light in tobacco. Plant Physiol..

[35] Sexton T.B., Christopher D.A., Mullet J.E. (1990). Light-induced switch in barley psbD-psbC promoter utilization: A novel mechanism regulating chloroplast gene expression. EMBO J..

[36] Nagashima A., Hanaoka M., Shikanai T., Fujiwara M., Kanamaru K., Takahashi H., Tanaka K. (2004). The multiple-stress responsive plastid sigma factor, SIG5, directs activation of the psbD Blue Light-Responsive Promoter (BLRP) in *Arabidopsis thaliana*. Plant Cell Physiol..

[37] Gamble P.E., Sexton T.B., Mullet J.E. (1988). Light-dependent changes in psbD and psbC transcripts of barley chloroplasts: Accumulation of two transcripts maintains psbD and psbC translation capability in mature chloroplasts. EMBO J..

[38] Yao W.B., Meng B.Y., Tanaka M., Sugiura M. (1989). An additional promoter within the protein-coding region of the psbD-psbC gene cluster in tobacco chloroplast DNA. Nucleic Acids Res..

[39] Chan P.P., Lowe T.M. (2019). tRNAscan-SE: Searching for tRNA genes in genomic sequences. Methods Mol. Biol..

[40] Chan P.P., Lin B.Y., Mak A.J., Lowe T.M. (2019). tRNAscan-SE 2.0: Improved detection and functional classification of transfer RNA genes. bioRxiv.

[41] Zhelyazkova P., Sharma C.M., Förstner K.U., Liere K., Vogel J., Börner T. (2012). The primary transcriptome of barley chloroplasts: Numerous noncoding RNAs and the dominating role of the plastid-encoded RNA polymerase. Plant Cell.

[42] Zhelyazkova P., Hammani K., Rojas M., Voelker R., Vargas-Suárez M., Börner T., Barkan A. (2012). Protein-mediated protection as the predominant mechanism for defining processed mRNA termini in land plant chloroplasts. Nucleic Acids Res..

[43] Kwok C.K., Ding Y., Tang Y., Assmann S.M., Bevilacqua P.C. (2013). Determination of in vivo RNA structure in low-abundance transcripts. Nat. Commun..

[44] Talkish J., May G., Lin Y., Woolford J.L., McManus C.J. (2014). Mod-seq: High-throughput sequencing for chemical probing of RNA structure. RNA.

[45] Wang Z., Tang K., Zhang D., Wan Y., Wen Y., Lu Q., Wang L. (2017). High-throughput m6A-seq reveals RNA m6A methylation patterns in the chloroplast and mitochondria transcriptomes of *Arabidopsis thaliana*. PLoS ONE.

[46] Burgess A.L., David R., Searle I.R. (2015). Conservation of tRNA and rRNA 5-methylcytosine in the kingdom Plantae. BMC Plant Biol..

[47] Song J., Yi C. (2017). Chemical modifications to RNA: A new layer of gene expression regulation. ACS Chem. Biol..

[48] Sun L., Xu Y., Bai S., Bai X., Zhu H., Dong H., Wang W., Zhu X., Hao F., Song C.-P. (2019). Transcriptome-wide analysis of pseudouridylation of mRNA and non-coding RNAs in *Arabidopsis*. J. Exp. Bot..

[49] Small I.D., Schallenberg-Rüdinger M., Takenaka M., Mireau H., Ostersetzer-Biran O. (2019). Plant organellar RNA editing: What 30 years of research has revealed. Plant J..

[50] Barkan A. (2011). Expression of plastid genes: Organelle-specific elaborations on a prokaryotic scaffold. Plant Physiol..

[51] Ruwe H., Kupsch C., Teubner M., Schmitz-Linneweber C. (2011). The RNA-recognition motif in chloroplasts. J. Plant Physiol..

[52] Hammani K., Bonnard G., Bouchoucha A., Gobert A., Pinker F., Salinas T., Giegé P. (2014). Helical repeats modular proteins are major players for organelle gene expression. Biochimie.

[53] Manavski N., Schmid L.-M., Meurer J. (2018). RNA-stabilization factors in chloroplasts of vascular plants. Essays Biochem..

[54] Jiang J., Chai X., Manavski N., Williams-Carrier R., He B., Brachmann A., Ji D., Ouyang M., Liu Y., Barkan A. (2019). An RNA chaperone-like protein plays critical roles in chloroplast mRNA stability and translation in *Arabidopsis* and *Maize*. Plant Cell.

[55] McDermott J.J., Civic B., Barkan A. (2018). Effects of RNA structure and salt concentration on the affinity and kinetics of interactions between pentatricopeptide repeat proteins and their RNA ligands. PLoS ONE.

[56] Takyar S., Hickerson R.P., Noller H.F. (2005). mRNA helicase activity of the ribosome. Cell.

[57] Horlitz M., Klaff P. (2000). Gene-specific trans-regulatory functions of magnesium for chloroplast mRNA stability in higher plants. J. Biol. Chem..

[58] Wang Z., Wang M., Wang T., Zhang Y., Zhang X. (2019). Genome-wide probing RNA structure with the modified DMS-MaPseq in *Arabidopsis*. Methods.

[59] Langmead B., Salzberg S.L. (2012). Fast gapped-read alignment with Bowtie 2. Nat. Methods.

[60] Morgan M., Pagès H., Obenchain V., Hayden N. (2020). Rsamtools: Binary alignment (BAM), FASTA, variant call (BCF), and tabix file import. R package version 2.2.3. http://bioconductor.org/packages/Rsamtools.

[61] Robin X., Turck N., Hainard A., Tiberti N., Lisacek F., Sanchez J.-C., Müller M. (2011). pROC: An open-source package for R and S+ to analyze and compare ROC curves. BMC Bioinform..

[62] Mathews D.H., Turner D.H., Watson R.M. (2016). RNA secondary structure prediction. Curr. Protoc. Nucleic Acid Chem..

[63] Lai D., Proctor J.R., Zhu J.Y.A., Meyer I.M. (2012). R-CHIE: A web server and R package for visualizing RNA secondary structures. Nucleic Acids Res..

[64] Darty K., Denise A., Ponty Y. (2009). VARNA: Interactive drawing and editing of the RNA secondary structure. Bioinformatics.

[65] Starmer J., Stomp A., Vouk M., Bitzer D. (2006). Predicting shine-dalgarno sequence locations exposes genome annotation errors. PLoS Comput. Biol..

