# Supplementary material for: Secondary Structure of Chloroplast mRNAs In Vivo and In Vitro
Source: Plants (Basel). 2020 Mar 4;9(3):323. doi: 10.3390/plants9030323 (PMC7154907; doi:10.3390/plants9030323)
Supplement: Supplementary file 1 [file plants-09-00323-s001.zip › plants-721614-supplementary-for conversion/plants-721614-supplementary-for conversion.pdf]

plants

MDPI

Supplementary

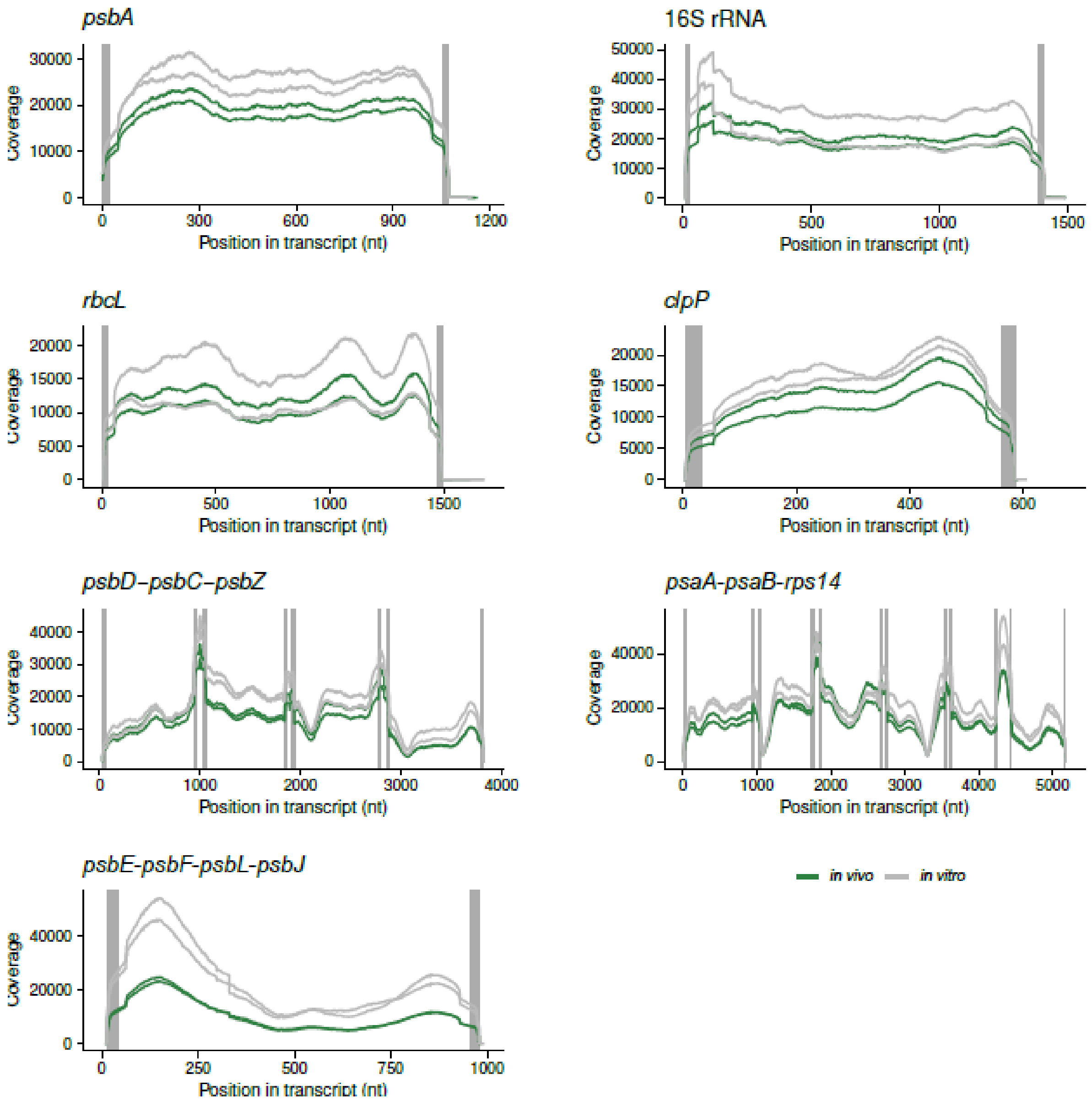

Supplemental Figure S1. Illumina reads' coverage of chloroplast transcripts in *in vivo* (green) and *in vitro* folded (grey) samples. Two biological replicates each are shown. Grey, vertical lines depict regions bound by the primers. These regions are excluded from the further analysis. Longer transcripts were amplified with several overlapping pairs of primers.

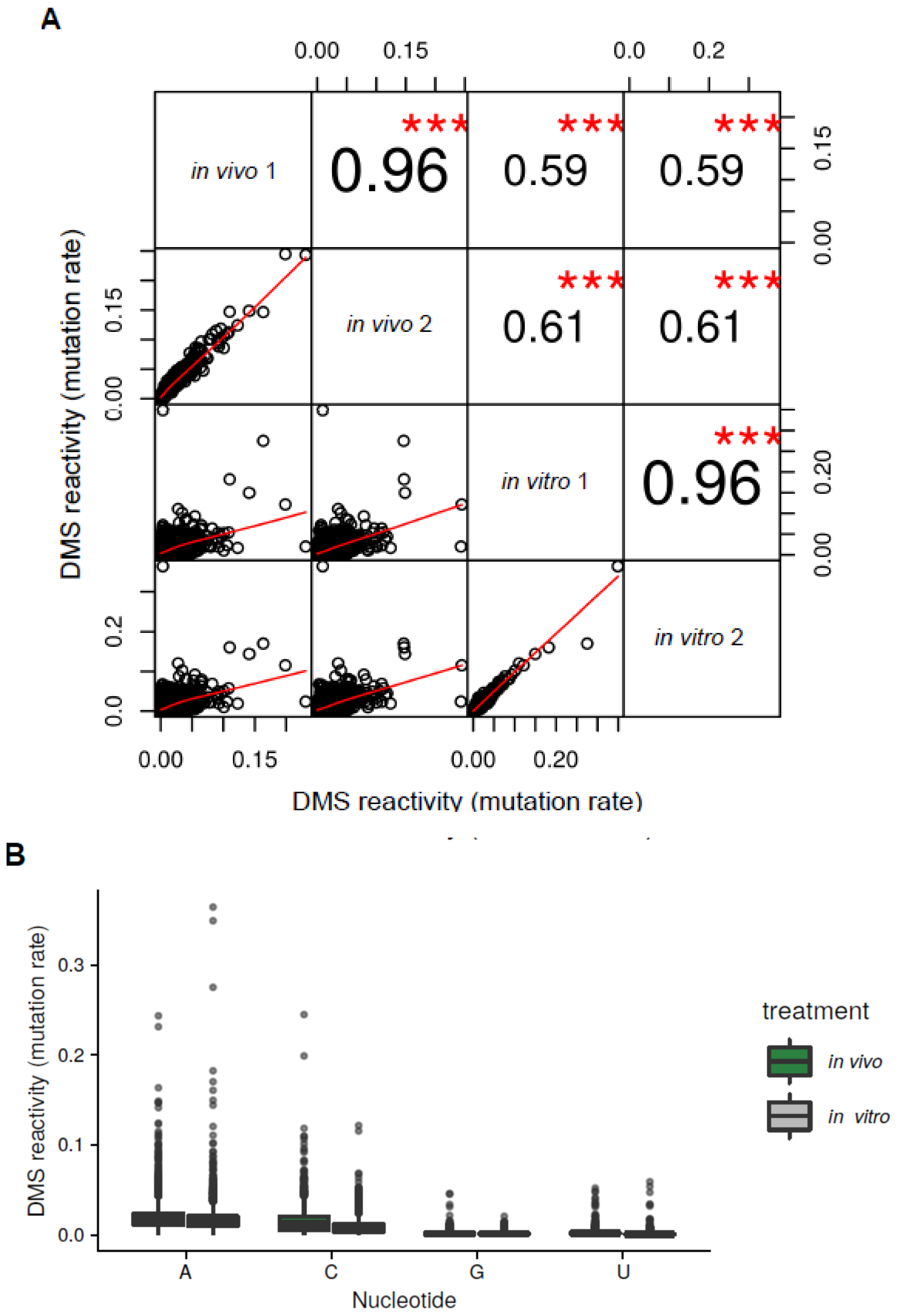

Supplemental Figure S2. Reproducibility of DMS reactivities. (**A**) Pair-wise comparisons of *in vivo* and *in vitro* samples. Spearman's *r* and *P* values (*** $P < 0.001$) are given. (**B**) DMS reactivity at all four nucleotides in all analyzed RNAs *in vivo* and *in vitro*. For the coverage, compare to Supplemental Figure S1.

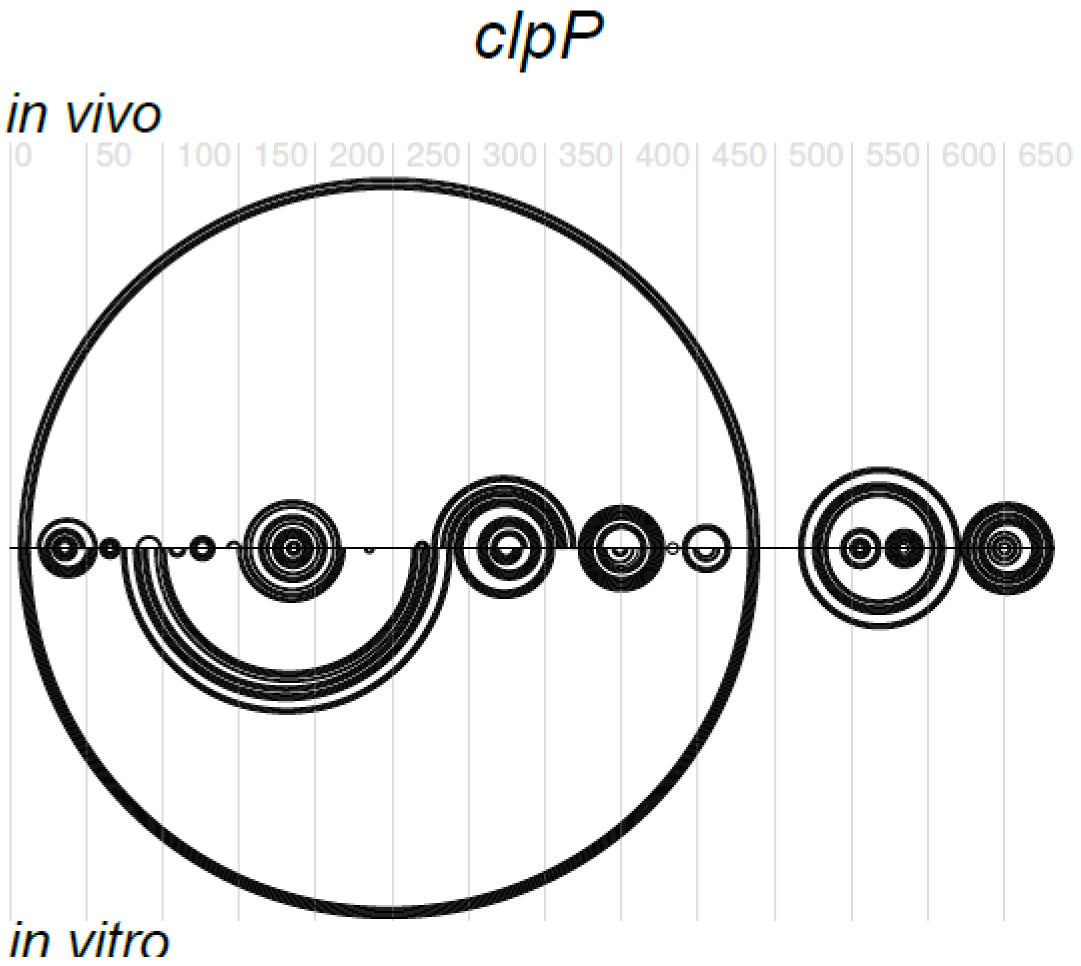

Supplemental Figure S3. Comparison of normalized DMS reactivities (of As and Cs) at all analyzed RNAs *in vivo* and in *vitro*.

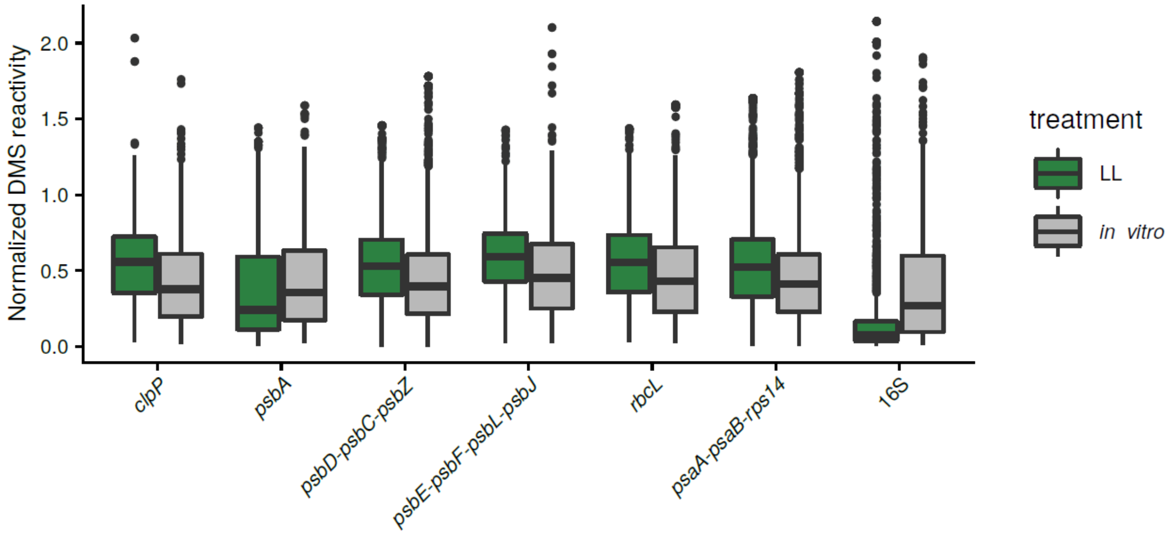

Supplemental Figure S4. Predicted mRNA secondary structures of *clpP in vivo* and of *in vitro* using DMS reactivities (at adenosines and cytidines) as constrains presented as arc-plots. The full-length transcript was used for the predictions.

Table S2. List of primers used in this study.

Name	Sequence 5′ → 3′	Comment
16S_8_MaP_F	GAGAGTTCGATCCTGGCTCA	
16S_1408_MaP_R	CCCTCCTTGCGGTTAAGGTA	Used in RT and in PCR
psbA_1_MaP_F	AACAAGCTCTCAATTATCTACT	
psbA_1070_MaP_R	ATAACTTCCATACCAAGGTT	
psbA_MaP_RP	TGTAGATGGAGCCTCAACAGCA	RT primer
rbcL_F2	TTGGCGAATCAAATATCATGGTC	
rbcL_R2	CTGCAAGATCACGTCCCTCA	
rbcL_MaP_RP	AGATTGAGCCGAGTGCAATTAAACT	RT primer
clpP_1_MaP_LP	ACGTTTCCACATCAAAGTGAAATAGAGA	
clpP_1_MaP_RP	ATCCCGTTCCATGTCTTCGGAT	Used in RT and in PCR
psbD_1_MaP_LP	TTGACAGATACATAAGGGCATGTACA	
psbD_1_MaP_RP	AACTAGGCGCAAAGAACCAACC	Used in RT and in PCR
psbD_2_MaP_LP	TGCTCGATCTGTTCAATTGCGA	
psbD_2_MaP_RP	AATACCGCCAAAGCCCAAAACT	Used in RT and in PCR
psbD_3_MaP_LP	GTAGGTCCTGGGGGAGAAGTTA	
psbD_3_MaP_RP	CGTGCCATAAATGACCCACGAA	Used in RT and in PCR
psbD_4_MaP_LP	CGTAGCTACTGAGATCAATGCAGT	
psbD_4_MaP_RP	AATTCAAGGAATGGGAGGGGGT	Used in RT and in PCR
psaA_1_MaP_LP	CCCCAGATCGACTTCCAGATCA	
psaA_1_MaP_RP	CGGTTAACCATAGACCCCCAGT	Used in RT and in PCR
psaA_2_MaP_LP	ACTTTATCCAAGTTTTGCTGAAGGAG	
psaA_2_MaP_RP	AACGCGAGCTACGAGCAAATAG	Used in RT and in PCR
psaA_3_MaP_LP	AAGTAGCTTTGTTACCTATTCCATTAGGA	
psaA_3_MaP_RP	GCCGGTTGACCAAAATGAGGAT	Used in RT and in PCR
psaA_4_MaP_LP	TGTTTCATGTAGCTTGGCAAGGA	
psaA_4_MaP_RP	CCATGAGCAAAAGCTCCTGTCA	Used in RT and in PCR
psaA_5_MaP_LP	ACCTGCTTATGCGTTCATAGCG	
psaA_5_MaP_RP	GTAGCCCAAACAAGATGCCCAA	Used in RT and in PCR
psaA_6_MaP_LP	GGATGGGTTACTTTTTATTGGCATTGG	
psaA_6_MaP_RP	AATGGTAAAGGGGCCTCATCGA	Used in RT and in PCR
psbE_MaP_LP	AATACCCTTGGTACTTTATTGACGATCT	
psbE_MaP_RP	CAAAGAAGGAAGAGTTGGGCCC	Used in RT and in PCR

Table S3. Table showing number of additional PCR cycles relative to *psbA* (25 cycles).

Transcript	Additional PCR Cycles
clpP	6
psbA	0
rbcL	1
16S	−2
psbD-psbC-psbZ	4
psaA-psaB-rps14	4
psbE-psbF-psbL-psbJ	3

© 2020 by the authors. Licensee MDPI, Basel, Switzerland. This article is an open access article distributed under the terms and conditions of the Creative Commons Attribution (CC BY) license (http://creativecommons.org/licenses/by/4.0/).
